# “When they say weed causes depression, but it’s your fav antidepressant”: Knowledge-aware attention framework for relationship extraction

**DOI:** 10.1371/journal.pone.0248299

**Published:** 2021-03-25

**Authors:** Shweta Yadav, Usha Lokala, Raminta Daniulaityte, Krishnaprasad Thirunarayan, Francois Lamy, Amit Sheth

**Affiliations:** 1 Wright State University, Dayton, Ohio, United States of America; 2 University of South Carolina, Columbia, South Carolina, United States of America; 3 Arizona State University, Tempe, Arizona, United States of America; 4 Mahidol University, Salaya, Thailand; King Abdullah University of Science and Technology, SAUDI ARABIA

## Abstract

With the increasing legalization of medical and recreational use of cannabis, more research is needed to understand the association between depression and consumer behavior related to cannabis consumption. Big social media data has potential to provide deeper insights about these associations to public health analysts. In this interdisciplinary study, we demonstrate the value of incorporating domain-specific knowledge in the learning process to identify the relationships between cannabis use and depression. We develop an end-to-end knowledge infused deep learning framework (Gated-K-BERT) that leverages the pre-trained BERT language representation model and domain-specific declarative knowledge source (Drug Abuse Ontology) to jointly extract entities and their relationship using gated fusion sharing mechanism. Our model is further tailored to provide more focus to the entities mention in the sentence through entity-position aware attention layer, where ontology is used to locate the target entities position. Experimental results show that inclusion of the knowledge-aware attentive representation in association with BERT can extract the cannabis-depression relationship with better coverage in comparison to the state-of-the-art relation extractor.

## 1 Introduction

Depression is one of the most common mental disorders in the United States. The 2017 US National Surveys on Drug Use and Health (NSDUH) indicated that approximately 13.2% (3.2 million) of adolescents aged 12 to 17-year-old and 7.1% (17.3 million) adults 18-year-old and older reported experiencing at least one major depressive episode [1]. Although the prevalence of depression in the US population, especially among young adults [2] has increased, and a variety of pharmacological treatments are available, a large proportion of individuals with depression delay seeking treatment or avoid it altogether [3]. According to the 2017 NSDUH data, an estimated 35% of adults and 60.1% of adolescents who had a major depressive episode did not receive treatment [1].

Current medical cannabis policies across the US do not include depression as a medical qualifying condition for medical cannabis use [4]. However, emerging research indicates that coping with depression is often reported as an important reason for cannabis use [5]. However, the potential causal relationship and directionality between cannabis use and depression remain uncertain [6]. There is a paucity of research on the topic and existing studies mainly focus on treatment centers data [7]. Furthermore, the on-going and rapid changes in the US cannabis legislative landscape along with the increased potency of cannabis over the past twenty years call for timely epidemiological monitoring of lay practices and therapeutic uses of cannabis products in order to assess the impact of policy changes, and identifying emerging issues and trends [8, 9].

In this context, social media platforms play an important role in uncovering experiences of individuals and their health-related knowledge [10, 11]. Social media data offers the possibility to indirectly collect information about those who do not receive treatment while having depression and using cannabis. Although user generated content area constitutes a rich source of unsolicited and unfiltered self-disclosures of attitudes and practices related to cannabis use [12–14], they have not been explored to derive insights about causal relationship between cannabis and depression.

Despite the recent improvements in Natural Language Processing (NLP) techniques, scientific literature utilizing NLP to investigate this type of relationship and/or focus on cannabis use remains sparse. Research has investigated the relation between cannabis use and psychosis based on Electronic Medical Records using NLP techniques [15, 16]. Basic NLP techniques were also used to assess the frequency of experienced effects and harms of different generations of synthetic cannabinoids in drug-focused web forums [17]. However, and to the best of our knowledge, no research using relation extraction has investigated the link existing between cannabis and depression based on social media data.

Therefore, this research aims to design a relation extraction method facilitating the identification of the causal relationship between cannabis and depression as expressed by cannabis users using Twitter data. While we acknowledge that correlations so derived are not to be confounded with causation, they do provide insights on potential hypotheses that can be explored through RCTs in the future.

We formulate this problem as the extraction of relationship between cannabis use and depression in terms of four possible relationships namely: *Reason*, *Effect*, *Addiction*, and *Ambiguous* (*c.f*. Table 1). Extracting relationships between any *concepts/slang-terms/synonyms/street-names* related to ‘*cannabis*’, and similarly those related to ‘*depression*’, requires a domain ontology. Here, we use Drug Abuse Ontology (DAO) [18, 19] a domain-specific hierarchical framework containing 315 entities (814 instances) and 31 relations defining drug-use and mental-health disorder concepts. The ontology has been utilized in analyzing web-forum content related to buprenorphine, cannabis, synthetic cannabinoid, and opioid-related data [17, 20–23]. The DAO included representations of mental health disorders and related symptoms that were developed following DSM-5 classification. These terms were collected from the medical literature related to substance use, abuse and addiction. In addition to medical terminology, DAO included commonly used lay and slang terms that were identified using prior clinical literature and social media-based studies on depressive symptomatology [24].

**Table 1:** Cannabis-depression Tweets and their relationships.

Relationship	Tweet
Reason	“-Not saying im cured, but i feel less depressed lately, could be my CBD oil supplement.”
	“I treat my depression with weed. I dont trust depression meds. I’ll stick to the all natural.”
Effect	“-People will smokeweed and be on antidepressants. It’s a clash!Weed is what is making you depressed.”
	“About to smoke a blunt of depression”
Addiction	“-The lack of weed in my life is depression as hell.”
	“i decided not to eat edibles n now i feel Depressed”
Ambiguous	“-People with an aversion to weed heavily are like intentionally depressed.”
	“i’m depressed drunk and on marijuana”

Here the text in the blue and red represents the cannabis and depression entities respectively.

The DAO was expanded using DSM-5 categories covering the most common mental health disorders by utilizing the study of [25] for improving data collection about mental health and cannabis use on Twitter. The lexicon for DSM-5 has been constructed by utilizing publicly available knowledge bases, namely, ICD-10 [26], SNOMED-CT [27], and DataMed [28], along with enriched Drug Abuse Ontology.

For entity and relationship extraction (RE) task, previous approaches generally adopt deep learning models [29–31], in particular, Convolutional Neural Network (CNN) [32, 33] and Bi-directional Long Short Term Memory (Bi-LSTM) [34–36] networks. However, Bi-LSTM/CNN model does not generalize well and performs poorly in limited supervision scenarios [37, 38]. Recently, several pre-trained language representation models have significantly advanced the state-of-the-art in various NLP tasks [39, 40]. BERT [41] is one of the powerful language representation models that has the ability to make predictions that go beyond the natural sentence boundaries [42]. Unlike CNN/LSTM model, language models benefit from the abundant knowledge from pre-training using self-supervision and have strong feature extraction capability. So we exploit the representation from BERT and CNN to achieve best of both the representations using novel gating fusion mechanism. Further, we tailored our model to capture the entities position information (using DAO knowledge) which is crucial for the RE as established in the prior research [43, 44].

We propose an end-to-end knowledge-infused deep learning framework (named, ***Gated-K-BERT***) based on widely adopted BERT language representation model and domain-specific DAO ontology to extract entities and their relationship. The proposed model has three modules: ***(1) Entity Locator***, which utilizes the DAO ontology to map the input word sequence to the entities mention in the ontology by computing the edit distance between the entity names (obtained from the DAO) and every n-gram token of the input sentence. ***(2) Entity Position-aware Module***, exploits the DAO to explicitly integrate the knowledge of entities in the model. This is done by encoding position sequence relative to the entities. Further, we make the attention layers aware of the positions of all entities in the sentence. ***(3) Encoding Module***, jointly leverages the distributed representation obtained from BERT and entity position-aware module using the shared gated fusion layer to learn the contextualized syntactic and semantic information which are complimentary to each other.

**Contributions**:

**(1)** In collaboration with domain experts, we introduce an annotation scheme to label the relationships between cannabis and depression entities to generate a gold standard cannabis-depression relationship dataset extracted using Twitter.

**(2)** We propose an end-to-end knowledge-infused neural model to extract cannabis/depression entities and predict the relationship between those entities. We exploited domain-specific DAO ontology which provides better coverage in entity extraction. We further augment the BERT model into knowledge-aware framework using gated fusion layer to learn the joint feature representation.

**(3)** We explored entity position-aware attention in the task to jointly leverages the distributed representation of word position relative to cannabis/depression mention and the attention mechanism.

**(4)** We evaluated our proposed model on real-world social media dataset. The experimental results shows that our model outperforms the state-of-the-art relation extraction techniques. We further analyzed that enhancing neural attention with entity position knowledge improves the performance of the model to predict the correct relationship between cannabis and depression over vanilla attention mechanism.

## 2 Related work

Based on the techniques, recent existing works can be broadly categorized into the following:

1. **Deep Learning (DL) framework**: Several DL approaches primarily based on CNN [45] and LSTM [29, 46, 47] techniques has been proposed for RE. A study by [48] develops a hybrid deep neural network model using Bi-Directional Gated Recurrent Neural Network (Bi-GRU), CNN, GRU, and Highway connection for classifying relations in SemEval 2010 and KBP-SF48 dataset. [49] exploited the dependency tree by utilizing Graph Convolutional Neural Network (GCN) to capture rich structural information that has been demonstrated for the RE task. [50] advanced the previous methods based on GCN by guiding the network through the attention mechanism. Another prominent work by [51] explores the adversarial learning to jointly extract entities and their relationship. To further enhance the performance of the DL models, various techniques [52, 53] has also exploited latent features in particular the entity position information in the DL framework.
2. **Pre-trained language representation model**: Models such as BERT, BioBERT [54], SciBERT [55], and XLNet [56] has shown the state-of-the-art performance on RE task. [57] adapted the BERT for the relation extraction and semantic role labeling task. [58] modified the BERT framework by constructing task-specific MASK that control the attention in last layers of the BERT. [59] also modified the original BERT architecture by introducing a structured prediction layer that is able to predict the multiple relations in one pass and make attention layers aware of the entities position.
3. **Knowledge-base framework**: Study by [60] saw the importance of external knowledge in improving the relation extraction from sentences. The study utilizes the parent-child relationships in Wikipedia and word cluster over unlabeled data into a global inference procedure using Integer Linear Programming (ILP). Experiments conducted on ACE-2004 dataset show that the use of background knowledge improved F-measure by 3.9%. A study by [61] uses the attention model to traverse a medical knowledge graph for entity pairs which assist in precise relation extraction. [62] jointly learn the word and entity embedding (obtained through the TransE) using the anchor context model to extract the relationship and the entities. Some of the other prominent work utilizing knowledge graph for relation extraction are [63–65].

## 3 Resource creation and annotation scheme

The corpus consists of tweets collected under the eDrugTrends project [66] that aimed to analyze trends in knowledge, attitudes, and behaviors related to the use of cannabis and synthetic cannabinoids on Web forums and Twitter. Tweets were collected from January 2017 to February 2019 using Twitter data processing, filtering, and aggregation framework available through the Twitris platform [67], which has been configured to collect tweets with relevant keywords selected by the epidemiologists in the team and adapted to perform appropriate analysis. Domain specialists (RD and FRL) in the team selected the most adapted keywords to identify both cannabis and depression based on the DAO and prior research [17, 20–24]. From the available corpus of over 100 million relevant tweets collected so far, we further filtered tweets using DAO based on Cannabis and Depression entities and their respective instances specifically defined by domain experts (substance use epidemiologist) for this context. From that filtered corpus, a sample of around 11,000 tweets was sent for expert annotation to a team of 3 substance use epidemiologist co-authors who have extensive experience in drug use, abuse, and addiction research. Further processing was done on this corpus based on the tweets lacking one of the key concepts related to cannabis/depression and 5,885 tweets were annotated finally. The annotation scheme is based on the following coding:

1. **Reason**: Cannabis is used to help/treat/cure depression.
2. **Effect**: Cannabis causes depression or makes symptoms worse.
3. **Addiction**: Lack of access to cannabis leads to depression, showing potential symptom of addiction.
4. **Ambiguous**: Implies other types or relationships, or too ambiguous/unclear to interpret.

The category “Addiction” is an intermediate between the first two as it indicates that feelings of depression would be resolved if one had access to cannabis (which relates to category 1) and also suggests potential presence of cannabis withdrawal symptoms, thus indicating that cannabis use could lead to depressive mood (as a part of withdrawal symptoms) [68]. Due to the brevity and ambiguity of information provided in the tweet content, the team decided to classify such cases as a separate category.

The sub-samples of tweets were coded independently by each coder and an inter-coder agreement was calculated. The team went through 3 iterations of coding, assessing and discussing, disagreement, and improving coding rules until an acceptable level of agreement was reached among coders (Cohen’s kappa of 0.80,(*c.f*. Table 2)) [69]. Tweets that were coded differently by two primary coders were reviewed by a third coder to resolve the disagreement. This yielded a dataset containing 5,885 tweets out of which **(1)** 3243 tweets are annotated as ‘Reason’ **(2)** 707 tweets are annotated as ‘Effect’. **(3)** 158 tweets are annotated as ‘Addiction’ **(4)** 1777 tweets are annotated as ‘Ambiguous’. The mean tweet text length is 148 tokens (median 74).

**Table 2:** Pairwise average annotator agreement using Cohen’s kappa between 4 annotators over 3 cycles.

	B	C	D
**A**	**0.83**	0.79	0.75
**B**	-	0.75	**0.86**
**C**	-	-	0.80

The university institutional review board (IRB) approved the study under Human Subjects Research Exemption 4 because it is limited to publicly available tweets. To protect anonymity, cited tweet content was modified slightly. We note that this dataset has some (inevitable) limitations: **(i)** the method only captures a sub-population of cannabis-depression related tweets in eDrugTrends campaign (i.e. those with terms defined in ontology), **(ii)** Tweets collected may not be a representative sample of the population as a whole, and **(iii)** there is no way to verify whether the tweets with self-reported cannabis related depression or cannabis related relief from depression are truthful. The team included researchers with extensive expertise in substance use epidemiology and community-based research, and they contributed to development of the annotated sample.

**Ethics**: Our project involves analysis of Twitter data that is publicly available and that has been anonymized. It does not involve any direct interaction with any individuals or their personally identifiable data. Thus, this study was reviewed by the Wright State University IRB and received an exemption determination.

## 4 Our proposed approach

**Fig 1:**
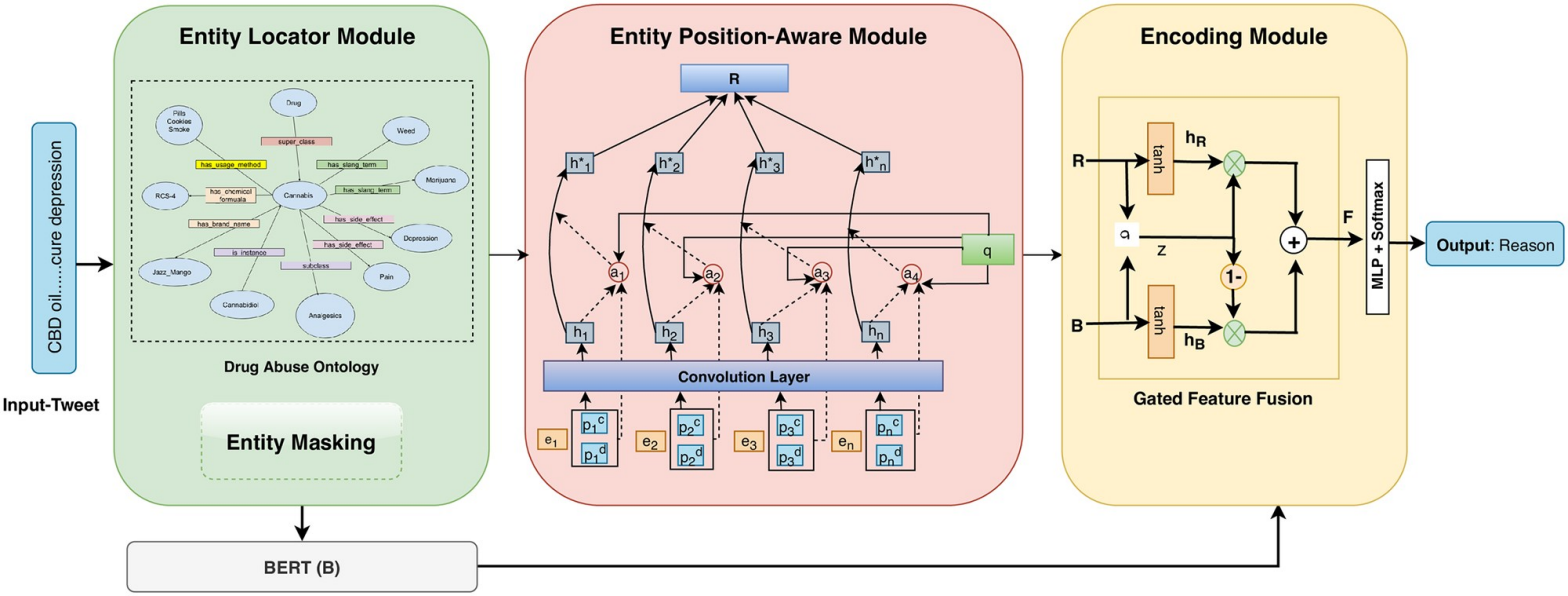
Proposed model architecture for cannabis-depression relation extraction. Input to the model is the tweet and output is the relationship between cannabis and depression entity.

In this study, a knowledge-infused RE framework, Gated Knowledge BERT (Gated-K-BERT) is used to identify relations between entities ‘*cannabis*’ and ‘*depression*’ in a tweet. Our framework (*c.f*. Fig 1) consists of three components discussed as follows:

### 4.1 Entity locator module

Let *S* be an input tweet containing the *n* words {*w*_1_, *w*_2_, …, *w*_*n*_}. Extracting relationships between any *concepts/slang-terms/synonyms/street-names* related to ‘*cannabis*’ and similarly those related to ‘*depression*’ require heavy dependency on the domain knowledge model. We used domain-specific DAO to map entities in a tweet to their parent concepts in the ontology by computing the edit distance between the entity names (obtained from the DAO) and every n-gram token of the input sentence. Since, DAO provides much better coverage on the entities, it is assume that entity name will be mention in the sentence.

Later, we perform masking on the extracted entities. The reason for masking is to explicitly provide the model with the entity information and also prevent a model from overfitting its predictions to specific entities. For instance, entities related to cannabis in a tweet are masked by ‘<*cannabis*>’. Similarly, entities related to depression are masked with ‘<*depression*>’. By this, we obtain a cannabis entity *c* and a depression entity *d* in the tweet, corresponding to two non-overlapping consecutive spans of length *k* and *l*: $S_{c}=\left\{w_{c_{1}},w_{c_{2}},\ldots ,w_{c_{k}}\right\}$ and $S_{d}=\left\{w_{d_{1}},w_{d_{2}},\ldots ,w_{d_{l}}\right\}$. In effect, this processing abstracts different lexical sequence in tweets to their meaning.

### 4.2 Entity position-aware module

This module is designed to infuse the knowledge of the entity mention in basic neural models to effectively capture the contextual information w.r.t the entities. The module consists of following three layers as:

#### 4.2.1 Position embedding layer

Inspired by the position encoding vectors used in [70, 71], we define a position sequence relative to the cannabis entity $\left\{p_{1}^{c},p_{2}^{c},\ldots ,p_{l}^{c}\right\}$, where

$$\begin{array}{c} p_{i}^{c}=\{\begin{array}{cc} i-c_{1} & i<c_{1}\\ 0 & c_{1}\leq i\leq c_{k}\\ i-c_{k} & i>c_{k} \end{array} \end{array}$$ (1)

Here, $p_{i}^{c}$ is the relative distance of token *w*_*i*_ to the cannabis entity and *c*_1_ and *c*_*k*_ are the beginning and end indices of the cannabis entity, respectively. In the same way, we computed the relative distance $p_{i}^{d}$ of token *w*_*i*_ to the depression entity. This provides two position sequences $p^{c}=\left\{p_{1}^{c},p_{2}^{c},\ldots ,p_{n}^{c}\right\}$ and $p^{d}=\left\{p_{1}^{d},p_{2}^{d},\ldots ,p_{n}^{d}\right\}$. Later, for each position in the sequence, an embedding is learned with an embedding layer to producing two position embedding vectors, $P^{c}=\left\{P_{1}^{c},P_{2}^{c},\ldots ,P_{n}^{c}\right\}$ for cannabis position embeddings and $P^{d}=\left\{P_{1}^{d},P_{2}^{d},\ldots ,P_{n}^{d}\right\}$, both sharing a position embedding matrix P respectively.

Further, we map each of the tokens from the input tweet *S* to the pre-trained word embedding matrix $E\in R^{V\times d}$ having the vocabulary size *V* and dimension *d*. We used FastText [72], a pre-trained word embedding. Word Embedding (Word2Vec) utilizes dense vectors to represent each word in the vocabulary by projecting into a continuous vector space and also captures both syntactic and semantic information associated with the words. However, in case of a tweet, incorrect spellings, slangs, and other word forms, are out-of-vocabulary (OOV) terms with respect to the word2vec model. In contrast, character-level embeddings have the ability to address the OOV issues by learning the vectors of character n-grams or parts of the words. FastText, unlike word2vec, is trained on the character-level corpus that enables the model to capture words that have similar meanings but different morphological word formations in a robust manner.

We represent the input tweet after applying the word embedding as *e* = {*e*_1_, *e*_2_, … *e*_*n*_}, where $e_{i}\in R^{d\times d}$. Finally each word *i* in the tweet *S* is represented as the concatenation of the word embedding and relative distance of position embedding with respect to cannabis and depression:

$$\begin{array}{c} x_{i}=e_{i}⊕P_{i}^{c}⊕P_{i}^{d} \end{array}$$ (2)

We denote the final representation of tweet as *x* = {*x*_1_, *x*_2_, … *x*_*n*_}. The word feature and position feature representations compose a position-aware representation.

#### 4.2.2 Convolution layer

A combined representation of word and position embedding sequence *x* is passed to the convolution layer, where filter $\text{F}\in R^{m\times d}$ is convoluted over the context window of *m* words for each tweet. In order to ensure that the output of the convolution layer is of the same length as input, we performed the necessary zero-padding on the input sequence *x*. We call the zero-padded input as $\bar{x}$.

$$\begin{array}{c} f_{i}^{m}=tanh\left(\text{F}.{\bar{x}}_{i:i+m-1}+b\right) \end{array}$$ (3)

where *tanh* is the non-linear activation function and *b* is a bias term. The feature map *f* is generated by applying a given filter **F** to each possible window of words in a tweet, Mathematically,

$$\begin{array}{c} f^{m}=\left[f_{1}^{m},f_{2}^{m},\ldots ,f_{n}^{m}\right] \end{array}$$ (4)

We apply different length of context window *m* ∈ *M*, where *M* is the set of context window length. Finally, we generate the hidden state *h*_*i*_ at time *i* as the concatenation of all the convoluted features by applying a different window size at time *i*.

#### 4.2.3 Entity position-aware attention layer

The intuition behind adding entity position-aware attention layer is to select relevant contexts over irrelevant ones [73]. This position-aware representation of entities in a tweet is further modulated by an ontology developed by domain experts. This enhancement enables us to selectively model attention and weigh entities in a tweet. The position-aware attention layer takes as an input *h*_1_, *h*_2_, *h*_3_, …‥*h*_*n*_ from the encoding module. We formulate an aggregate vector **q** mathematically as follows:

$$\begin{array}{c} \text{q}=\frac{1}{n}\sum_{i=1}^{n}h_{i} \end{array}$$ (5)

The vector **q**, thus, stores the global, semantic, and syntactic information contained in a tweet. With the aggregate vector, we compute attention weight *a*_*i*_ for each hidden state *h*_*i*_ as

$$\begin{array}{c} u_{i}=v^{T}\;\text{tanh}\left(W_{h}h_{i}+W_{q}q+W_{c}P_{i}^{c}+W_{d}P_{i}^{d}\right) \end{array}$$ (6)

$$\begin{array}{c} \alpha_{i}=\frac{exp(u_{i})}{\sum_{j=1}^{n}exp\left(u_{j}\right)} \end{array}$$ (7)

where, $W_{h},W_{q}\in R^{d_{a}\times d_{h}};W_{c},W_{d}\in R^{d_{a}\times d_{p}};V\in R^{d_{a}}$ are parameters of the network, where *d*_*h*_ is the dimension of the hidden states, *d*_*p*_ is the dimension of position embedding, *d*_*a*_ is the size of attention vector. After applying the attention, the final tweet representation *R* is computed as

$$\begin{array}{c} R=\sum_{j=1}^{n}\alpha_{j}h_{j} \end{array}$$ (8)

### 4.3 Encoding module

In the encoding module, we aim to obtain the semantic and task-specific contextualized representation of the tweet. We leverage the joint representation through BERT language representation model and Entity position-aware module.

Owing to its effective word and sentence level representation, BERT provide a task-agnostic architecture that has achieved state-of-the-art status for various NLP tasks [39, 74]. We use the pre-trained BERT model having 12 Transformer layers (*L*), each having 12 heads for self-attention and hidden dimension 768. The input to the BERT model is the tweet *S* = {*w*_1_, *w*_2_, …, *w*_*n*_}. It returns the hidden state representation of each Transformer layer. Formally,

$$\begin{array}{c} H_{b}^{1},H_{b}^{2},\ldots ,H_{b}^{L}=BERT\left(\left[w_{1},w_{2},\ldots ,w_{n}\right]\right) \end{array}$$ (9)

where, $H_{i}\in R^{n\times h_{b}}$ and *h*_*b*_ is the dimension of the hidden state representation obtained from BERT. We masked the representation of [CLS] and [SEP] tokens with zero. We obtained the tweet representation via BERT model as follows:

$$\begin{array}{c} B=\frac{1}{n}\sum_{j=1}^{n}H_{b}^{L-1}\left[j,:\right] \end{array}$$ (10)

In our experiments, the representation obtained from the second last (*L* − 1) Transformer layer achieved the best performance on the task. The representation obtained from the last Transformer layer is too close to the target functions (i.e., masked language model and next sentence prediction tasks) during pre-training of BERT, therefore may be biased to those targets. We also experiment with the [CLS] token representation obtained from BERT but that could not perform well in our experimental setting.

#### 4.3.1 Gated feature fusion

The feature generated from CNN and BERT capture different aspect from the data. These features need to be used carefully to make most out of them. The joint feature obtained from concatenation or other arithmetic operations (sum, difference, min, max etc) often results in the poor joint representation. To mitigate this issue, we propose a gated feature fusion technique, which learn the most optimal way to join both the feature representation using a neural gate. This gate learn what information from CNN or BERT feature representation to keep or exclude during the network training. The gating behaviour is obtained through a *sigmoid* activation which range between 0 and 1. We learn the joint representation *F* using the gated fusion as follows:

$$\begin{array}{c} \begin{array}{cc} h_{R} & =tanh(W_{R}.R)\\ h_{B} & =tanh(W_{B}.B)\\ g & =sigmoid(W_{g}.\left[R⊕B\right])\\ F & =g*h_{R}+\left(1-g\right)*h_{B} \end{array} \end{array}$$ (11)

where, *W*_*R*_, *W*_*B*_ and *W*_*g*_ are the parameters. Finally, the joint feature representation *F* fed into a single layer feed-forward network with *softmax* function to classify the tweet into one of the relation classes, *Y* = {‘*reason*’, ‘*effect*’, ‘*addiction*’, ‘*ambiguous*’}. More, formally,

$$\begin{array}{c} p\left(\hat{y}|S\right)=softmax\left(W.F+a\right) \end{array}$$ (12)

where $\hat{y}\in Y$, *W* is a weight matrix and *a* is the bias.

## 5 Experimental setup and results

Here, we present results on the cannabis-depression RE task. Thereafter, we will provide technical interpretation of the results followed by domain interpretation of the results. We have chosen models’ hyper-parameters using 5-fold cross validation on entire dataset. The convolutional layer used filters of lengths 2, 3 and 4 and stride of length 1. The optimal feature size is turned out to be 128. We use Adam [75] as our optimization method with a learning rate of 0.001. Hidden size of feed forward in relation classification layer is set to 100. The position embedding is set to 100 for position-aware attention model. The optimal value of *d*_*a*_ is found to be 50 in all the experiment. We use the Adadelta [76] optimization algorithm to update the parameters in each epochs. As a regularizer, we use dropout [77] with a probability of 0.3.

### 5.1 Results

The dataset utilized in our experiment is described in Section-3. We used Recall, Precision and F_1_-Score to evaluate our proposed task against state-of-the-art relation extractor. As a baseline model, we used ***BERT***, ***BioBERT***, ***BERTweet*** [78] and its several variation such as:

**BERT_PE_**: We extend the BERT with the position information (relative distance of the current word w.r.t cannabis/depression entities) obtained through ontology, as a position embedding along with the BERT embedding.

**BERT_PE+PA_**: We introduced additional component to the BERT_PE_ model by deploying position-aware attention mechanism.

Table 3 summarizes the performance of our model over the baselines. Our proposed model significantly outperforms the state-of-the-art baselines on all the evaluation metrics. In comparison with the BERT & BioBERT, our model achieves the absolute improvement of 2.9% & 3.69% F_1_-Score respectively. Second, the results shows that infusing entity knowledge in the form of entity position-aware encoding with attention can assist in better relation classification.

**Table 3:** Performance comparison our proposed model with the baselines methods.

Models	Techniques Used	Cannabis-Depression RE		
		Precision	Recall	F_1_-Score
Baseline 1	BERT	64.49	63.22	63.85
Baseline 2	BioBERT	63.97	62.15	63.06
Baseline 3	BERTweet	65.34	62.70	63.99
Baseline 4	BERT_PE_	60.64	56.51	58.50
Baseline 5	BERT_PE+PA_	65.41	65.25	64.50
**Proposed Approach**	**Gated-K-BERT**	**66.41**	**67.10**	**66.75**

Among all the BERT-based approaches, we found that BERT_PE_ did not perform well. Thus merely including position-aware encoding in the BERT framework does not help model to capture the entities information. This may be due to the inbuilt position embedding layer in the BERT model which treats the explicit position encoding as a noise. Further, our observation shows that BioBERT did not generalize well for our task in comparison to the BERT with minor reduction of 0.79% absolute F_1_-Score. Although BioBERT is trained on huge corpus of biomedical literature (PubMed & PMC), however data being noisy hampered to performance.

Interestingly, adding the entity position information in the form of the attention (BERT_PE+PA_) boosted the model performance. We report the performance absolute improvements of 0.92%, 2.03%, and 0.65% Precision, Recall, and F_1_-Score points in comparison to the BERT model. This shows that position encoding and position attention when used collectively can assist in capturing complementary features. Our final analysis reveals that solely concatenating two representation (CNN+BERT) may not be enough to capture how much information is required from both of these representations. Our method, which introduces the gated fusion mechanism can address this problem as validated by the improved F_1_-Score (c.f. Table 4).

**Table 4:** Performance comparison of our proposed model with/without gated fusion mechanism.

Model	Precision	Recall	F_1_-Score
**Proposed Model (-) Gated Fusion**	67.35	64.07	65.67
**Proposed Model (+) Gated Fusion**	66.41	67.10	66.75

We also reported the class-wise performance of our proposed model in Table 5. The performance of the classes ‘Effect’ and ‘Addiction’ comparatively lower than other classes. It is because the classes ‘Effect’ and ‘Addiction’ have less samples (707, 158) in the dataset which inhibits to learn and generalize the model, in contrast to other classes. Also, in the real-life the explicit expression of being addicted to cannabis after depression can rarely be identified with a single tweets. It requires more contextual knowledge of users history of at-least 2 weeks tweets to capture the implicit sense of this class.

**Table 5:** Class-wise performance of the proposed model.

Classes	Precision	Recall	F1-Score
Reason	77.17	80.48	78.79
Effect	50.53	35.84	41.93
Addiction	37.34	27.74	31.84
Ambiguous	55.51	58.75	57.08

### 5.2 Ablation study

To analyze the impact of various component of our model, we perform the ablation study (c.f. Table 6) by removing one component from the proposed model and evaluate the performance. Results show that excluding BERT from the model significantly drop the recall of the model by 5.27%, and F_1_-Score by 3.16%. This shows that contextualized representation is highly necessary for the cannabis-depression classification task.

**Table 6:** Ablation Study: The value within the bracket shows the absolute decrements in the model by removing the respective component.

Model	Precision	Recall	F_1_-Score
**Proposed Model Gated(CNN+PE+PA+BERT)**	66.41	67.10	66.75
•**BERT**	65.54 (0.87↓)	61.83 (5.27↓)	63.59 (3.16↓)
•**Position-aware Attention**	64.94 (1.47↓)	64.63 (2.47↓)	64.79 (1.96↓)
•**Position Embedding**	65.68 (0.73↓)	65.18(1.92↓)	65.43 (1.32↓)
•**CNN**	60.55 (5.86↓)	57.26(9.84↓)	58.86 (7.89↓)

We further observed that entity position-aware attention is highly crucial for improving the precision of the model. We report a reduction of 1.47% in terms of precision after excluding the position attention as the model component.

Similarly, removing the position encoding from the input layer also lead to a reduced performance. While, excluding convolution layer from the model leads to significant drop in precision, recall, and F_1_-Score by 5.56%, 9.84%, and 7.89% respectively. Thus, we show that every component in the model is beneficial for the cannabis-depression relation extraction task.

We also evaluated our proposed entity locator module (based on edit distance) over simple string matching technique. The results show that though the string-matching technique performs well (94.36% F-Score), there are some cases like ‘*smokin chronic*’, and ‘*marijuana candies*’ that are not handled correctly by basic string-matching technique, since DAO contains the standard entity names as ‘*smoking chronic*’, and ‘*marijuana candy*’. Unlike in our proposed approach, DAO based entity locator module is build upon the domain-specific ontology which contain medical and slang terms related to substance use, abuse, and addiction. Further, as edit distance method allow the soft-string matching (with the insert, delete, and update operation) within the threshold, it captures to match the ill-formed entities (‘*smokin chronic*’ to ‘*smoking chronic*’) more accurately over string-matching method. Our DAO based entity locator is more focused and accurate (97.12% F-Score) in spotting entities related to cannabis and depression in tweets.

We also compared the position-aware attention over vanilla (word-level) attention discuss in [79]. We called it *vanilla attention* as it weighs each word equally regardless of the corresponding entities. Given the hidden states *h*_1_, *h*_2_, *h*_3_, …‥*h*_*n*_, in word-level attention, first the hidden state *h*_*t*_ of each time step *t* is transformed into *u*_*t*_ using one-layer feed-forward network. Thereafter, the importance *α*_*t*_ of each token representation is computed using the *softmax* layer. Formally,

$$\begin{array}{c} \begin{array}{cc} u_{t} & =tanh(W_{u}h_{t}+b_{u})\\ \alpha_{t} & =\frac{exp(u_{t}^{⊤}v)}{\sum_{i=1}^{i=n}exp\left(u_{i}^{⊤}v\right)} \end{array} \end{array}$$ (13)

where, *W*_*u*_ *v*, and *b*_*u*_ are weight matrix, context vector and bias respectively. The final tweet representation *R* is computed as

$$\begin{array}{c} R=\sum_{i=1}^{n}\alpha_{j}h_{j} \end{array}$$ (14)

The results (*c.f*. Table 7) shows that the position aware attention achieve the better performance (an improvement of 2.45 in F1-Score) over the vanilla attention.

**Table 7:** Performance comparison of our proposed model with position-aware attention over vanilla attention.

Proposed Model	Precision	Recall	F_1_-Score
**with Position-aware Attention**	66.41	67.10	66.75
**with Vanilla Attention**	66.43	64.91	64.30

### 5.3 Domain-specific analysis

To assess the performance on our model, we examined a set of correctly and incorrectly classified, tweets and came up with the following observations:

- **Correctly classified tweets generally contained clear relationship words**: For example, the following two tweets were correctly classified as expressing cannabis use to treat depression:
  “weed really helps my depression so much! i get less irritable, laugh, and so much more and people think it as the devil! f*** you mean”;
  “*marijuana is seriously my best friend rn. it helps me sooo much with my depression and anxiety*.”
  Both tweet contained word “help” that often times is used to convey a meaning indicating usage of a drug for the treatment of a certain condition.
- The following correctly classified example represented a case where relationship indicating “treat” was expressed with a word “for”:
  “*I was forced to tell my family i have a medical for weed bc someone been ratting me out, try explaining medical marijuana for depression to a traditional thinking family, i wanna die*”.
- Similarly, the following tweet were correctly classified as expressing situations where cannabis use is causing depression and/or making it worse:
  “*me @ me when i realize weed is making me depressed but i keep smoking*”.
  Both tweets contained clear relationship word expressing causation “make/making”.
- **The incorrectly classified tweets generally were more ambiguous and/or contained implied meanings**. For example, the following tweet was labeled as expressing “cannabis use to treat depression” while our model classified it as “ambiguous”:
  “*depression is hitting insufferable levels rn and hot damn i could use some weed*.”
  This is an example, where relationship is implied, and there are no clear relationship word expressed in the text.
- The same misclassification occurred with the following tweet:
  “*me: wow i think im depressed i should really go to therapy: doesnt do any of that and instead uses weed to increase the dopamine in my brain*.”
  In this case, the expression “used weed to increase the dopamine…” implies use of marijuana to improve mood (in this cases depressive mood).
  Because DAO did not contain similar colloquial expressions to indicate depressive mood, our model failed to correctly classify this tweet.

Overall, our model performs better than state-of-the-art algorithm in distinguishing depression as a result of cannabis use and cannabis use as a self-medication for depression. In turn, this model will help future works to collect relevant information specific to the behaviors, attitudes and knowledge of users who use cannabis to palliate their depression as well as information on the Twitter users who suffer from depression because of their previous cannabis usage.

## 6 Limitations

Limitations are noted. First, our work does not distinguish Cannabidiol (CBD) use from general cannabis use. This is of importance as several studies suggest that CBD could reduce anxiety and potentially depression [80]. Although users tend to be more specific regarding whether they are consuming CBD specifically rather than other form of cannabis, future research on the topic of cannabis and depression using social media data needs to integrate this distinction. Second, although the goal of this study was to design a robust algorithm able to differentiate the causal relationship between cannabis and depression as expressed by Twitter users, our work did not aim to establish the objective causal “directionality” in between cannabis and depression (i.e., is cannabis causing depression or is cannabis a potential treatment for depression?). Third, the model has been trained on Twitter data that are rather short (280 characters maximum) form of text, and may not be as performing on other text format (e.g., blog, web forums pages).

## 7 Conclusion

This research explored a new dimension of social media in identifying and distinguishing relationships between cannabis and depression. We introduced a state-of-the-art knowledge-aware attention framework that jointly leverages knowledge from the domain-specific DAO, DSM-5 in association with BERT for cannabis-depression RE task. Further, our result and domain analysis help us find associations of cannabis use with depression. In order to establish a more accurate and precise Reason-Effect relationship between cannabis and depression from social media sources, our future study would take targeted user profiles in real-time and study the exposure of the user to cannabis over time informing public health policy.

## Supporting information

S1 Data (TSV)

## References

[1] Major Depression; 2019. https://www.nimh.nih.gov/health/statistics/major-depression.shtml.

[2] Weinberger AH, Gbedemah M, Martinez AM, Nash D, Galea S, Goodwin RD. Trends in depression prevalence in the USA from 2005 to 2015: widening disparities in vulnerable groups. Psychol Med. 2018;48(8):1308–1315. 10.1017/S0033291717002781 29021005

[3] Young AS, Klap R, Shoai R, Wells KB. Persistent depression and anxiety in the United States: prevalence and quality of care. Psychiatr Serv. 2008;59(12):1391–1398. 10.1176/ps.2008.59.12.1391 19033165 PMC3086068

[4] Bridgeman MB, Abazia DT. Medicinal Cannabis: History, Pharmacology, And Implications for the Acute Care Setting. P T. 2017;42(3):180–188. 28250701 PMC5312634

[5] Lankenau SE, Kioumarsi A, Reed M, McNeeley M, Iverson E, Wong CF. Becoming a medical marijuana user. International Journal of Drug Policy. 2018;52:62–70. 10.1016/j.drugpo.2017.11.018 29247863 PMC5808850

[6] Womack SR, Shaw DS, Weaver CM, Forbes EE. Bidirectional associations between cannabis use and depressive symptoms from adolescence through early adulthood among at-risk young men. Journal of studies on alcohol and drugs. 2016;77(2):287–297. 10.15288/jsad.2016.77.287 26997187 PMC4803661

[7] Gukasyan N, Strain EC. Relationship between cannabis use frequency and major depressive disorder in adolescents: findings from the national survey on drug use and health 2012–2017. Drug and alcohol dependence. 2020;208:107867. 10.1016/j.drugalcdep.2020.107867 31958677 PMC7039755

[8] Room R. Legalizing a market for cannabis for pleasure: Colorado, Washington, Uruguay and beyond; 2014. 10.1111/add.12355 24180513

[9] Volkow ND, Baler RD, Compton WM, Weiss SRB. Adverse health effects of marijuana use. N Engl J Med. 2014;370(23):2219–2227. 10.1056/NEJMra1402309 24897085 PMC4827335

[10] Corazza O, Assi S, Simonato P, Corkery J, Bersani FS, Demetrovics Z, et al. Promoting innovation and excellence to face the rapid diffusion of novel psychoactive substances in the EU: the outcomes of the ReDNet project. Hum Psychopharmacol. 2013;28(4):317–323. 10.1002/hup.2299 23881879

[11] Burns L, Roxburgh A, Bruno R, Van Buskirk J. Monitoring drug markets in the Internet age and the evolution of drug monitoring systems in Australia. Drug Test Anal. 2014;6(7-8):840–845. 10.1002/dta.1613 24574080

[12] Cavazos-Rehg PA, Zewdie K, Krauss MJ, Sowles SJ. “No High Like a Brownie High”: A Content Analysis of Edible Marijuana Tweets. Am J Health Promot. 2018;32(4):880–886. 10.1177/0890117116686574 29214836

[13] Daniulaityte R, Lamy FR, Smith GA, Nahhas RW, Carlson RG, Thirunarayan K, et al. “Retweet to Pass the Blunt”: Analyzing Geographic and Content Features of Cannabis-Related Tweeting Across the United States. J Stud Alcohol Drugs. 2017;78(6):910–915. 10.15288/jsad.2017.78.910 29087826 PMC5668996

[14] Lamy FR, Daniulaityte R, Zatreh M, Nahhas RW, Sheth A, Martins SS, et al. “You got to love rosin: Solventless dabs, pure, clean, natural medicine.” Exploring Twitter data on emerging trends in Rosin Tech marijuana concentrates. Drug Alcohol Depend. 2018;183:248–252. 10.1016/j.drugalcdep.2017.10.039 29306816 PMC5803369

[15] Patel R, Wilson R, Jackson R, Ball M, Shetty H, Broadbent M, et al. Association of cannabis use with hospital admission and antipsychotic treatment failure in first episode psychosis: an observational study. BMJ open. 2016;6(3). 10.1136/bmjopen-2015-009888 26940105 PMC4785290

[16] Irving J, Patel R, Oliver D, Colling C, Pritchard M, Broadbent M, et al. Using natural language processing on electronic health records to enhance detection and prediction of psychosis risk. Schizophrenia bulletin. 2020. 10.1093/schbul/sbaa126 PMC7965059 33025017

[17] Lamy FR, Daniulaityte R, Nahhas RW, Barratt MJ, Smith AG, Sheth A, et al. Increases in synthetic cannabinoids-related harms: Results from a longitudinal web-based content analysis. International Journal of Drug Policy. 2017;44:121–129. 10.1016/j.drugpo.2017.05.007 28578250 PMC5545681

[18] Cameron D, Smith GA, Daniulaityte R, Sheth AP, Dave D, Chen L, et al. PREDOSE: a semantic web platform for drug abuse epidemiology using social media. J Biomed Inform. 2013;46(6):985–997. 10.1016/j.jbi.2013.07.007 23892295 PMC3844051

[19] Drug Abuse Ontology | NCBO BioPortal;. http://bioportal.bioontology.org/ontologies/DAO.

[20] Lokala U, Lamy FR, Daniulaityte R, Sheth A, Nahhas RW, Roden JI, et al. Global trends, local harms: availability of fentanyl-type drugs on the dark web and accidental overdoses in Ohio. Comput Math Organ Theory. 2018; p. 1–12. 10.1007/s10588-018-09283-0 PMC7311101 32577089

[21] Cameron D, Smith GA, Daniulaityte R, Sheth AP, Dave D, Chen L, et al. PREDOSE: a semantic web platform for drug abuse epidemiology using social media. Journal of biomedical informatics. 2013;46(6):985–997. 10.1016/j.jbi.2013.07.007 23892295 PMC3844051

[22] Kumar R, Yadav S, Daniulaityte R, Lamy F, Thirunarayan K, Lokala U, et al. eDarkFind: Unsupervised Multi-view Learning for Sybil Account Detection. In: Proceedings of The Web Conference 2020; 2020. p. 1955–1965.

[23] Lamy FR, Daniulaityte R, Barratt MJ, Lokala U, Sheth A, Carlson RG. Listed for sale: analyzing data on fentanyl, fentanyl analogs and other novel synthetic opioids on one cryptomarket. Drug and alcohol dependence. 2020;213:108115. 10.1016/j.drugalcdep.2020.108115 32585419 PMC7736148

[24] Mowery DL, Park YA, Bryan C, Conway M. Towards automatically classifying depressive symptoms from Twitter data for population health. In: Proceedings of the Workshop on Computational Modeling of People’s Opinions, Personality, and Emotions in Social Media (PEOPLES); 2016. p. 182–191.

[25] Gaur M, Kursuncu U, Alambo A, Sheth A, Daniulaityte R, Thirunarayan K, et al. Let Me Tell You About Your Mental Health!: Contextualized Classification of Reddit Posts to DSM-5 for Web-based Intervention. In: Proceedings of the 27th ACM International Conference on Information and Knowledge Management; 2018. p. 753–762.

[26] Organization WH, et al. The ICD-10 classification of mental and behavioural disorders: clinical descriptions and diagnostic guidelines. World Health Organization; 1992.

[27] Whetzel PL, Noy NF, Shah NH, Alexander PR, Nyulas C, Tudorache T, et al. BioPortal: enhanced functionality via new Web services from the National Center for Biomedical Ontology to access and use ontologies in software applications. Nucleic acids research. 2011;39(suppl_2):W541–W545. 10.1093/nar/gkr469 21672956 PMC3125807

[28] Chen X, Gururaj AE, Ozyurt B, Liu R, Soysal E, Cohen T, et al. DataMed–an open source discovery index for finding biomedical datasets. Journal of the American Medical Informatics Association. 2018;25(3):300–308. 10.1093/jamia/ocx121 29346583 PMC7378878

[29] Yadav S, Ekbal A, Saha S, Kumar A, Bhattacharyya P. Feature assisted stacked attentive shortest dependency path based Bi-LSTM model for protein–protein interaction. Knowledge-Based Systems. 2019;166:18–29. 10.1016/j.knosys.2018.11.020

[30] Srivastava A, Ekbal A, Saha S, Bhattacharyya P, et al. A recurrent neural network architecture for de-identifying clinical records. In: Proceedings of the 13th international conference on natural language processing; 2016. p. 188–197.

[31] Yadav S, Ekbal A, Saha S, Bhattacharyya P. Deep learning architecture for patient data de-identification in clinical records. In: Proceedings of the clinical natural language processing workshop (ClinicalNLP); 2016. p. 32–41.

[32] Lin Y, Shen S, Liu Z, Luan H, Sun M. Neural relation extraction with selective attention over instances. In: Proceedings of the 54th Annual Meeting of the Association for Computational Linguistics (Volume 1: Long Papers); 2016. p. 2124–2133.

[33] Ekbal A, Saha S, Bhattacharyya P, et al. A deep learning architecture for protein-protein interaction article identification. In: 2016 23rd International Conference on Pattern Recognition (ICPR). IEEE; 2016. p. 3128–3133.

[34] Lee J, Seo S, Choi YS. Semantic Relation Classification via Bidirectional LSTM Networks with Entity-Aware Attention Using Latent Entity Typing. Symmetry. 2019;11(6):785. 10.3390/sym11060785

[35] Yadav S, Ramteke P, Ekbal A, Saha S, Bhattacharyya P. Exploring Disorder-Aware Attention for Clinical Event Extraction. ACM Transactions on Multimedia Computing, Communications, and Applications (TOMM). 2020;16(1s):1–21. 10.1145/3372328

[36] Yadav S, Ekbal A, Saha S, Bhattacharyya P. A unified multi-task adversarial learning framework for pharmacovigilance mining. In: Proceedings of the 57th Annual Meeting of the Association for Computational Linguistics; 2019. p. 5234–5245.

[37] Chen C, Bai W, Davies RH, Bhuva AN, Manisty CH, Augusto JB, et al. Improving the generalizability of convolutional neural network-based segmentation on CMR images. Frontiers in cardiovascular medicine. 2020;7:105. 10.3389/fcvm.2020.00105 32714943 PMC7344224

[38] Liu P, Qiu X, Huang X. Recurrent neural network for text classification with multi-task learning. arXiv preprint arXiv:160505101. 2016.

[39] Maillard J, Clark S, Yogatama D. Jointly learning sentence embeddings and syntax with unsupervised tree-lstms. Natural Language Engineering. 2019;25(4):433–449. 10.1017/S1351324919000184

[40] Akbik A, Bergmann T, Vollgraf R. Pooled contextualized embeddings for named entity recognition. In: Proceedings of the 2019 Conference of the North American Chapter of the Association for Computational Linguistics: Human Language Technologies, Volume 1 (Long and Short Papers); 2019. p. 724–728.

[41] Devlin J, Chang MW, Lee K, Toutanova K. BERT: Pre-training of Deep Bidirectional Transformers for Language Understanding. In: Proceedings of the 2019 Conference of the North American Chapter of the Association for Computational Linguistics: Human Language Technologies, Volume 1 (Long and Short Papers). Minneapolis, Minnesota: Association for Computational Linguistics; 2019. p. 4171–4186. Available from: https://www.aclweb.org/anthology/N19-1423.

[42] Lin C, Miller T, Dligach D, Bethard S, Savova G. A BERT-based universal model for both within-and cross-sentence clinical temporal relation extraction. In: Proceedings of the 2nd Clinical Natural Language Processing Workshop; 2019. p. 65–71.

[43] Zhou D, Miao L, He Y. Position-aware deep multi-task learning for drug–drug interaction extraction. Artificial intelligence in medicine. 2018;87:1–8. 10.1016/j.artmed.2018.03.001 29559249

[44] He Z, Chen W, Li Z, Zhang M, Zhang W, Zhang M. SEE: Syntax-aware entity embedding for neural relation extraction. In: Thirty-Second AAAI Conference on Artificial Intelligence; 2018.

[45] Liu C, Sun W, Chao W, Che W. Convolution neural network for relation extraction. In: International Conference on Advanced Data Mining and Applications. Springer; 2013. p. 231–242.

[46] Miwa M, Bansal M. End-to-End Relation Extraction using LSTMs on Sequences and Tree Structures. In: Proceedings of the 54th Annual Meeting of the Association for Computational Linguistics (Volume 1: Long Papers); 2016. p. 1105–1116.

[47] Ningthoujam D, Yadav S, Bhattacharyya P, Ekbal A. Relation extraction between the clinical entities based on the shortest dependency path based LSTM. arXiv preprint arXiv:190309941. 2019.

[48] Liang D, Xu W, Zhao Y. Combining word-level and character-level representations for relation classification of informal text. In: Proceedings of the 2nd Workshop on Representation Learning for NLP; 2017. p. 43–47.

[49] Wu F, Souza A, Zhang T, Fifty C, Yu T, Weinberger K. Simplifying Graph Convolutional Networks. In: Chaudhuri K, Salakhutdinov R, editors. Proceedings of the 36th International Conference on Machine Learning. vol. 97 of Proceedings of Machine Learning Research. Long Beach, California, USA: PMLR; 2019. p. 6861–6871. Available from: http://proceedings.mlr.press/v97/wu19e.html.

[50] Guo Z, Zhang Y, Lu W. Attention Guided Graph Convolutional Networks for Relation Extraction. In: Proceedings of the 57th Annual Meeting of the Association for Computational Linguistics. Florence, Italy: Association for Computational Linguistics; 2019. p. 241–251. Available from: https://www.aclweb.org/anthology/P19-1024.

[51] Bekoulis G, Deleu J, Demeester T, Develder C. Adversarial training for multi-context joint entity and relation extraction. In: Proceedings of the 2018 Conference on Empirical Methods in Natural Language Processing. Brussels, Belgium: Association for Computational Linguistics; 2018. p. 2830–2836. Available from: https://www.aclweb.org/anthology/D18-1307.

[52] Choi SP. Extraction of protein–protein interactions (PPIs) from the literature by deep convolutional neural networks with various feature embeddings. Journal of Information Science. 2018;44(1):60–73. 10.1177/0165551516673485

[53] Peng Y, Lu Z. Deep learning for extracting protein-protein interactions from biomedical literature. In: BioNLP 2017; 2017. p. 29–38.

[54] Lee J, Yoon W, Kim S, Kim D, So C, Kang J. BioBERT: a pre-trained biomedical language representation model for biomedical text mining. Bioinformatics (Oxford, England). 2020;36(4):1234–1240. 10.1093/bioinformatics/btz682 31501885 PMC7703786

[55] Beltagy I, Lo K, Cohan A. SciBERT: A Pretrained Language Model for Scientific Text. In: Proceedings of the 2019 Conference on Empirical Methods in Natural Language Processing and the 9th International Joint Conference on Natural Language Processing (EMNLP-IJCNLP); 2019. p. 3606–3611.

[56] Yang Z, Dai Z, Yang Y, Carbonell J, Salakhutdinov RR, Le QV. XLNet: Generalized Autoregressive Pretraining for Language Understanding. Advances in Neural Information Processing Systems. 2019;32:5753–5763.

[57] Shi P, Lin J. Simple BERT Models for Relation Extraction and Semantic Role Labeling. arXiv preprint arXiv:190405255. 2019.

[58] Xue K, Zhou Y, Ma Z, Ruan T, Zhang H, He P. Fine-tuning BERT for joint entity and relation extraction in Chinese medical text. In: 2019 IEEE International Conference on Bioinformatics and Biomedicine (BIBM). IEEE; 2019. p. 892–897.

[59] Wang H, Tan M, Yu M, Chang S, Wang D, Xu K, et al. Extracting Multiple-Relations in One-Pass with Pre-Trained Transformers. In: Proceedings of the 57th Annual Meeting of the Association for Computational Linguistics; 2019. p. 1371–1377.

[60] Chan YS, Roth D. Exploiting background knowledge for relation extraction. Proceedings of the 23rd International Conference on. 2010.

[61] Wen D, Liu Y, Yuan K, Si S, Shen Y. Attention-Aware Path-Based Relation Extraction for Medical Knowledge Graph. In: Smart Computing and Communication. Springer International Publishing; 2018. p. 321–331.

[62] Distiawan B, Weikum G, Qi J, Zhang R. Neural Relation Extraction for Knowledge Base Enrichment. In: Proceedings of the 57th Annual Meeting of the Association for Computational Linguistics; 2019. p. 229–240.

[63] Li J, Huang G, Chen J, Wang Y. Dual CNN for Relation Extraction with Knowledge-Based Attention and Word Embeddings. Computational intelligence and neuroscience. 2019;2019. 10.1155/2019/6789520 31396271 PMC6664687

[64] Zhou H, Lang C, Liu Z, Ning S, Lin Y, Du L. Knowledge-guided convolutional networks for chemical-disease relation extraction. BMC bioinformatics. 2019;20(1):260. 10.1186/s12859-019-2873-7 31113357 PMC6528333

[65] Li P, Mao K, Yang X, Li Q. Improving Relation Extraction with Knowledge-attention. In: Proceedings of the 2019 Conference on Empirical Methods in Natural Language Processing and the 9th International Joint Conference on Natural Language Processing (EMNLP-IJCNLP); 2019. p. 229–239.

[66] eDrugTrends; 2019. https://medicine.wright.edu/citar/edrugtrends.

[67] Sheth A, Jadhav A, Kapanipathi P, Lu C, Purohit H, Smith AG, et al. Chapter title: Twitris-a system for collective social intelligence. Encyclopedia of social network analysis and mining. 2014. 10.1007/978-1-4614-6170-8_345

[68] Budney AJ, Hughes JR, Moore BA, Vandrey R. Review of the validity and significance of cannabis withdrawal syndrome. American journal of Psychiatry. 2004;161(11):1967–1977. 10.1176/appi.ajp.161.11.1967 15514394

[69] McHugh ML. Interrater reliability: the kappa statistic. Biochem Med. 2012;22(3):276–282. 10.11613/BM.2012.031 23092060 PMC3900052

[70] Collobert R, Weston J, Bottou L, Karlen M, Kavukcuoglu K, Kuksa P. Natural language processing (almost) from scratch. Journal of Machine Learning Research. 2011;12(Aug):2493–2537.

[71] Zeng D, Liu K, Lai S, Zhou G, Zhao J. Relation Classification via Convolutional Deep Neural Network. In: Proceedings of COLING 2014, the 25th International Conference on Computational Linguistics: Technical Papers. Dublin, Ireland: Dublin City University and Association for Computational Linguistics; 2014. p. 2335–2344. Available from: https://www.aclweb.org/anthology/C14-1220.

[72] Bojanowski P, Grave E, Joulin A, Mikolov T. Enriching Word Vectors with Subword Information. arXiv preprint arXiv:160704606. 2016.

[73] Zhang Y, Zhong V, Chen D, Angeli G, Manning CD. Position-aware attention and supervised data improve slot filling. In: Proceedings of the 2017 Conference on Empirical Methods in Natural Language Processing; 2017. p. 35–45.

[74] Hewitt J, Manning CD. A structural probe for finding syntax in word representations. In: Proceedings of the 2019 Conference of the North American Chapter of the Association for Computational Linguistics: Human Language Technologies, Volume 1 (Long and Short Papers); 2019. p. 4129–4138.

[75] Kingma D, Ba J. Adam: A method for stochastic optimization. arXiv preprint arXiv:14126980. 2014.

[76] Zeiler MD. ADADELTA: An Adaptive Learning Rate Method. CoRR. 2012;abs/1212.5701.

[77] Hinton GE, Srivastava N, Krizhevsky A, Sutskever I, Salakhutdinov RR. Improving neural networks by preventing co-adaptation of feature detectors. arXiv preprint arXiv:12070580. 2012.

[78] Nguyen DQ, Vu T, Nguyen AT. BERTweet: A pre-trained language model for English Tweets. In: Proceedings of the 2020 Conference on Empirical Methods in Natural Language Processing: System Demonstrations; 2020. p. 9–14.

[79] Yang Z, Yang D, Dyer C, He X, Smola A, Hovy E. Hierarchical attention networks for document classification. In: Proceedings of the 2016 Conference of the North American Chapter of the Association for Computational Linguistics: Human Language Technologies; 2016. p. 1480–1489.

[80] de Mello Schier AR, de Oliveira Ribeiro NP, Coutinho DS, Machado S, Arias-Carrión O, Crippa JA, et al. Antidepressant-like and anxiolytic-like effects of cannabidiol: A chemical compound of Cannabis sativa. CNS & Neurological Disorders-Drug Targets (Formerly Current Drug Targets-CNS & Neurological Disorders). 2014;13(6):953–960. 24923339 10.2174/1871527313666140612114838

